# Interaction of gold nanoparticles with proteins and cells

**DOI:** 10.1088/1468-6996/16/3/034610

**Published:** 2015-06-18

**Authors:** Pengyang Wang, Xin Wang, Liming Wang, Xiaoyang Hou, Wei Liu, Chunying Chen

**Affiliations:** 1 School of Materials and Architectural Engineering, Guizhou Normal University, Guiyang, People’s Republic of China; 2 CAS Key Lab for Biomedical Effects of Nanomaterials and Nanosafety, Institute of High Energy Physics and National Center for Nanoscience and Technology, Chinese Academy of Science, Beijing, People’s Republic of China; 3 Jiangsu Key Laboratory of Biological Cancer Therapy, Xuzhou Medical College, Xuzhou, People’s Republic of China

**Keywords:** gold nanoparticles, protein corona, biomedical effect, cellular response, cytotoxicity

## Abstract

Gold nanoparticles (Au NPs) possess many advantages such as facile synthesis, controllable size and shape, good biocompatibility, and unique optical properties. Au NPs have been widely used in biomedical fields, such as hyperthermia, biocatalysis, imaging, and drug delivery. The broad application range may result in hazards to the environment and human health. Therefore, it is important to predict safety and evaluate therapeutic efficiency of Au NPs. It is necessary to establish proper approaches for the study of toxicity and biomedical effects. In this review, we first focus on the recent progress in biological effects of Au NPs at the molecular and cellular levels, and then introduce key techniques to study the interaction between Au NPs and proteins. Knowledge of the biomedical effects of Au NPs is significant for the rational design of functional nanomaterials and will help predict their safety and potential applications.

## 1. Introduction

**Figure 1.**
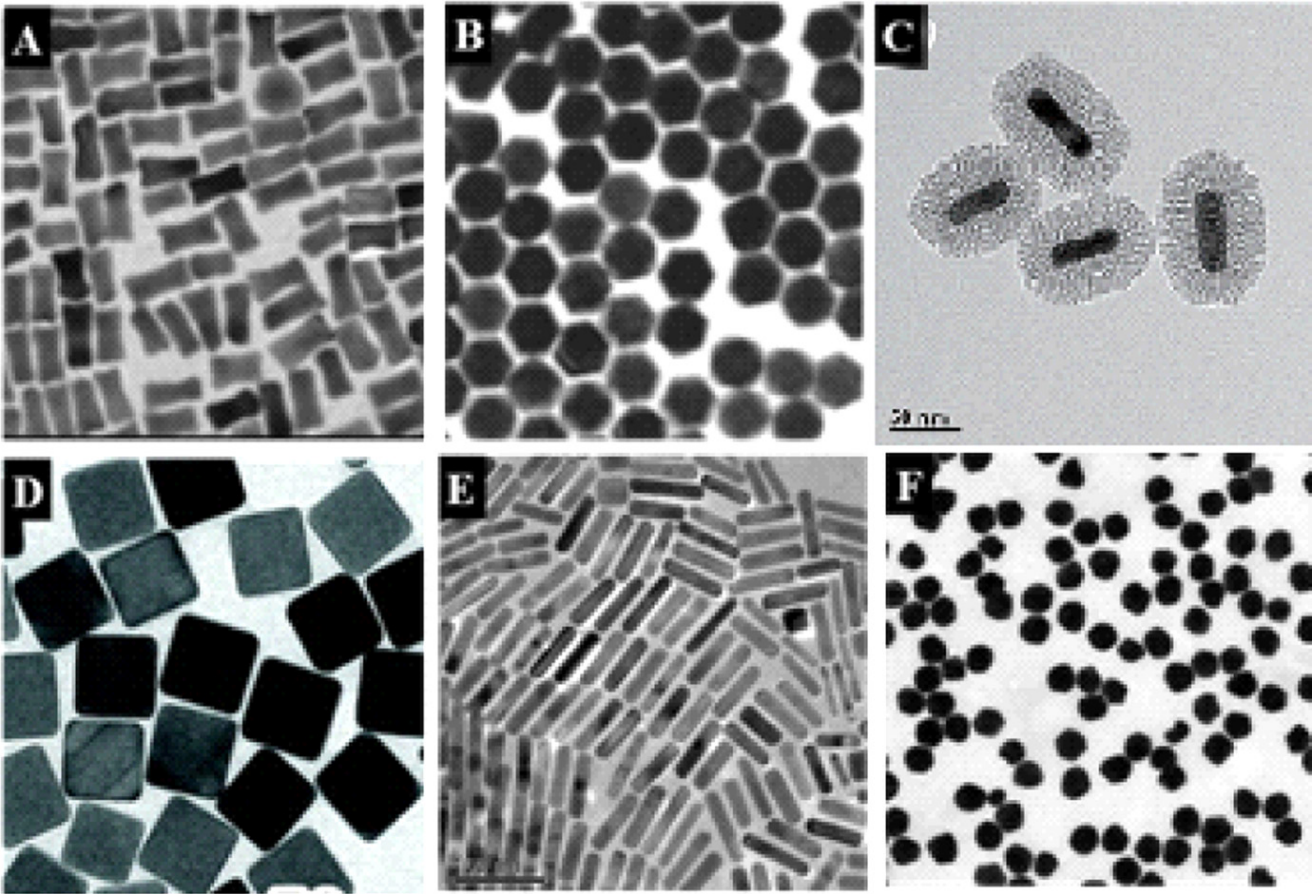
Various kinds of gold nanoparticles. (a) Gold nanobones. (b) Gold nanohoneycombs. Reprinted with permission from [130]. Copyright 2004 American Chemical Society. (c) Mesoporous silica-coated gold nanorods (Au@SiO_2_). Reprinted with permission from [10]. Copyright 2012 WILEY-VCH Verlag GmbH & Co. KGaA, Weinheim. (d) Gold nanocages. Reprinted with permission from [131]. Copyright 2007, rights managed by Nature Publishing Group. (e) Gold nanorods. Reprinted with permission from [79]. Copyright 2010 Elsevier Ltd. All rights reserved. (f) Gold nanospheres. Reprinted with permission from [132]. Copyright 2013 WILEY-VCH Verlag GmbH & Co. KGaA Weinheim.

Gold nanoparticles (Au NPs) are easy to prepare; they can have controllable shape and size (figure 1) with good biocompatibility, and optical properties such as surface enhanced Raman scattering (SERS) [1], surface plasmon resonance (SPR) [2], and two-photon luminescence (TPL) [3]. With these properties, Au NPs thus have widespread prospective applications in biomedical fields including imaging [4, 5], hyperthermia [6–10], drug and gene delivery [11–14], and biocatalysis [15–17]. For safe and efficient applications, much attention has been paid to the biomedical effects of Au NPs. The properties of shape, size, and surface chemistry play important roles in mediating the physiological behaviors of Au NPs: blood circulation [18], targeting [19], distribution [20], translocation [21], metabolism [22], clearance [23], and inflammation [24] *in vivo* and cellular pathways *in vitro* [25]. The study of long-term and short-term biological effects of Au NPs will contribute to understanding the biological behaviors and predicting nanotoxicity [26]. It is thus important to understand the potential risks or possible biomedical effects of Au NPs to human beings.

When Au NPs are exposed to biological fluids, proteins and other biomolecules are easily adsorbed onto the surface to form a protein ‘corona’ around Au NPs, which reduces the surface free energy of Au NPs [27]. Formation of a corona may change structures of adsorbed proteins [28, 29] and may also eliminate the physiological functions of proteins, which leads to the loss of original targeting capabilities [30], induces various cellular responses including inflammatory responses, increased lysosomal permeability, activated caspase-related pathways, or even apoptosis [31–35]. Therefore, understanding Au NP and protein interactions serves as a starting point to study the biological effects of Au NPs.

When Au NPs are injected into the body, they will interact with multiple types of cells, especially macrophages[36], endothelial cells [37], monocytes [38], and lymphocytes [39, 40]. In physiological conditions, Au NPs can be internalized, trafficked, stored, or secreted by cells [31, 41, 42]. At cellular levels, they may induce oxidation stress and cell apoptosis [43], trigger inflammatory responses [44], and mediate cell adhesion, migration [45], proliferation [46], and differentiation [47]. The sizes of Au NPs are at the nanoscale compared to those of proteins, intracellular components, or organelles [48]. Therefore, NP–cell interactions are a complicated interfacial process in space and time. State-of-the-art analytical techniques will be helpful in revealing the dynamic processes and potential mechanisms. Herein, we summarize the recent progress on the interaction of Au NPs with proteins and cells.

## 2. Au NP–protein interactions

Au NPs can enter the human body in different ways, among which the main exposure routes include inhalation, oral administration, intravenous injection, and dermal exposure [49]. Once Au NPs enter the body, they contact various biological molecules such as proteins, lipid, polysaccharides, and nucleic acids. Ubiquitous proteins are able to interact with Au NPs to form protein corona that influences the distribution of Au NPs in different organs or tissues [50].

### 2.1. The formation of corona

As we know, serum/plasma contains more than ten thousand kinds of protein. Because NPs have high surface free energy, in order to decrease NP surface energy [51], many kinds of protein can be adsorbed on the surface of Au NPs with distinct binding affinities [52, 53]. When exposed to biological fluids or microenvironments, Au NPs can contact and adsorb a variety of proteins such as ubiquitin [54, 55], serum albumin [56, 57], tumor necrosis factor [58, 59], cytochrome C [29], fibrinogen [60], or polypeptides [61]. Usually, the high abundance proteins first arrive at and adsorb on the surface of NPs, but they will be eventually replaced by high-affinity proteins to form NP–protein complexes [62]. Corona can be roughly divided into two types, hard and soft corona. Hard corona means that proteins are bound to the surface durably and tightly. In contrast, soft corona indicates that the proteins are less tightly bound to the surface, which is dynamic and will exchange with proteins in the media with time [63].

During the processes, chemical or physical adsorption takes part in the formation of protein corona. Coordination, hydrogen bonding, van der Waals forces, electrostatic and hydrophobic interactions, steric hindrance etc play important roles in driving the binding of proteins to Au NPs [64–67]. For instance, when bovine serum albumin (BSA) interacts with Au NPs, the disulfide bonds of BSA adsorb on the surface of Au NPs via at least 12 Au–S bonds [65]. In contrast, ubiquitin is a small, cysteine-free protein bound to citrate-coated Au NPs mainly via short-range, non-electrostatic interactions, such as hydrogen bonds, where the NH group binds to the central carboxylate group of surface citrate on NPs. Experimental approaches (nuclear magnetic resonance and circular dichroism) and computer simulations at multiple levels (*ab initio* quantum mechanics, classical molecular dynamics and Brownian dynamics) revealed the interaction details [55]. When citrate-modified Au NPs met lysozyme, the interaction induced the aggregation of proteins in physiological conditions. The authors used SERS, cryo-transmission electron microscope (TEM), and UV–visible spectroscopy to characterize the processes and found that the breakage of S–S bonds to form Au–S bonds changes in the conformation of lysozyme on the surface of Au NPs, induces protein unfolding, forms protein–Au nanoparticle assemblies, and produces protein aggregates [68].

The interaction of Au NPs with proteins may change the natural properties of both NPs and proteins. Importantly, the interaction can induce some physiological changes, including the configuration of bound proteins [28, 29, 60], activation of complement [69, 70], blood clotting [70], and aggregation of proteins [71, 72]. For example, when fibrinogens interact with poly(acrylic acid)-coated Au NPs, the protein will unfold and expose its cryptic peptide, specifically interact with the Mac-1 receptor, activate inflammation, and finally cause NF-*κ*B-dependent cytokine release [28].

### 2.2. Factors to mediate the protein corona composition

#### 2.2.1. NP size

The size of Au NPs influences the adsorbed amounts of protein on the surface. The reason is that the size of the NPs determines the curvature of NPs that have different protein binding constants. Poly(acrylic acid) (PAA)-coated Au NPs have negative charges on the surface and their sizes range from 7 nm to 22 nm (7, 10, 12, 15, 17, 19, and 22 nm). The binding affinity of Au NPs to fibrinogen increases with the size of Au NPs [60]. A similar study also showed that Au NPs (sized at 5, 10, 20, 30, 60, 80, and 100 nm) can interact with multiple proteins in blood including albumin, fibrinogen, globulin, histone, and insulin in a size-dependent manner. The authors used the efficiency of fluorescence quenching of plasma proteins to study the correlation of NP size to binding association constant. They found that Au NPs with increased sizes have stronger capability of binding to plasma proteins. They also showed that the adsorbed proteins undergo conformational change and the thickness of the adsorbed protein layer (size of Au NPs <50 nm) progressively increases with NP size in biological media [73]. Another team used gel electrophoresis and a combination of matrix-assisted laser desorption/ionization and time-of-flight mass spectrometry to quantitatively analyze and identify the mouse serum proteins adsorbed on 5, 15 and 80 nm phosphine stabilized Au NPs with negative surface charges. They found that smaller Au NPs have lower protein adsorption than larger Au NPs, because the former have a higher curvature that reduced the protein binding capacity [74].

#### 2.2.2. Hydrophilicity and hydrophobicity

Hydrophilicity or hydrophobicity of Au NPs are crucial factors to mediate the composition and amount of protein adsorption. After modification with high densities of hydrophilic polyethylene glycols (PEGs), Au NPs have a high capacity to resist the adsorption of plasma proteins such as complement components. Complement components are well known to be involved in the clearance of various NPs by macrophages when they are recognized by complement receptors. Therefore, these Au NPs have a long lifetime during blood circulation as they circumvent cellular uptake by reticuloendothelial systems (RES) [75]. In addition, stripe-like domains can be formed on the surface of Au NPs with a binary mixture of hydrophobic and hydrophilic thiolated ligand molecules. These stripe-like domains produce heterogeneities for both surface ligands with different charged functional groups (i.e., with COO^−^ or 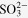 terminals) and hydrophobicity on the NPs. Based on dynamic light scattering (DLS), circular dichroism (CD) spectroscopy, fluorescence quenching, and isothermal titration calorimetry (ITC), the authors studied the adsorption of BSA onto three sulfonated alkanethiols (11-mercapto-1-undecanesulfonate, MUS)-type and two negatively charged MUS substituted with mercaptopropionic acid (MPA)-type Au NPs. Many positively charged side chains of the BSA surface will likely provide binding sites to interact with the negatively charged ligands on the NP surface, while sites with many nonpolar side chains may contact tthe nonpolar stripes on MUS and 1-octanethiol (MUS-OT) NPs. By tuning the ratio of hydrophobic and hydrophilic molecules, the surface structural heterogeneity thus serves as a new tunable property in modulating the conformation and the orientations of the adsorbed proteins [76]. Moreover, for the same nanoparticles, when compared with their hydrophilic counterparts, hydrophobic nanoparticles can adsorb more proteins from plasma [77].

#### 2.2.3. Surface chemistry

The surface of Au NPs is usually modified with electrolytes such as citrate, hexadecyl trimethyl ammonium bromide (CTAB), sodium polystyrene sulfonate (PSS), polyethylene diallyl dimethyl amine hydrochloride (PDDAC), etc in order to increase their dispersibility [78]. The surface modification provides enough net charges on the Au NPs, which may produce electrostatic attraction to the oppositely charged functional groups in proteins. For example, positively charged PDDAC-gold nanorods (Au NRs) will adsorb more serum proteins than negatively charged PSS-Au NRs based on SDS-PAGE results [79].

**Figure 2.**
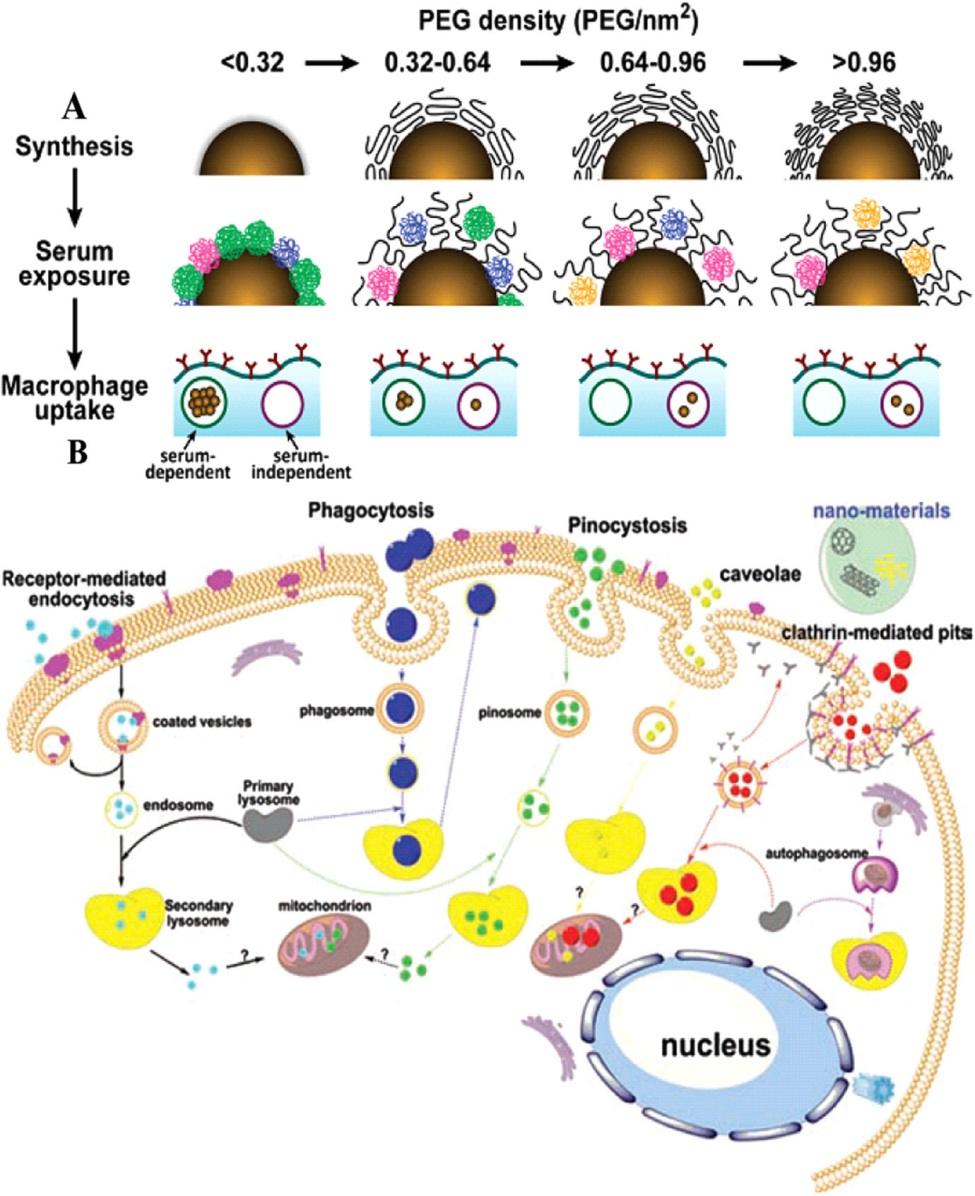
(a) Docking density of the modified PEG influencing the amount of protein adsorbed on the surface of Au NPs. PEG-modified density then determines the Au NP amount by macrophage uptake. Reprinted with permission from [80]. Copyright 2012 American Chemical Society. (b) Cellular uptake pathways for NPs: intracellular trafficking processes and possible endocytosis pathways. Reprinted with permission from [42]. Copyright 2011 WILEY-VCH Verlag GmbH & Co. KGaA, Weinheim.

In addition, serum protein corona bound on the surface of PEG-grafted Au NPs can be characterized by liquid chromatography tandem mass spectrometry, and more than 70 proteins on the surface have been identified. The amount of protein adsorption depends on the size of the Au NPs and the density of grafted PEG. Specifically, the greater the PEG coating density (molecular weight: 5 kDa), the smaller the amount of adsorbed proteins on the surface [80] (figure 2(a)). Cui *et al* studied the interaction of different Au NPs (modified by citrate, thioglycolic acid, cysteine, polyethylene glycol (molecular weight: 5 kDa and 2 kDa)) with serum proteins (bovine serum, albumin, transferrin (TRF) and fibrinogen (FIB)). The results showed that there is negligible protein adsorption on the surface of PEG-Au NPs. Fibrinogen can induce NP aggregation when it interacts with other non-PEG-coated Au NPs, while citrate or thioglycolic acid-coated Au NPs are capable of adsorbing TRF and BSA to form a 6–8 nm thickness corona [81]. The molecular weight of PEG ligands on Au NPs also influenced the bound proteins. The authors used PEG with various molecular weights (2, 5, 10, and 20 kDa) to modify 30 nm Au NPs and they found that the amount of adsorbed proteins and kinetics of protein binding are negatively related with the molecular weight of PEG [82].

### 2.3. Characterization of protein corona

To some extent, the interplay between Au NPs and proteins not only changes the properties of Au NPs, but also induces structural change in the adsorbed proteins. As a result, Au NPs acquire a new biological identity and mediate the properties and physiological functions of protein–NP complexes. Relevant analytical techniques are thus necessary to study dynamic processes for NP–protein interactions and to characterize the properties of the NP–protein complex, which are crucial to understanding the potential effects of Au NPs on cells and mechanisms.

One hot topic about protein corona is about the formation and evolution process of protein corona and the major composition of adsorbed proteins. A series of analysis methods and techniques such as optical absorption spectroscopy, TEM and DLS, have been used to characterize the protein composition and structure. TEM and DLS are widely used to measure the thickness of the protein corona on NPs in dried samples and in an aqueous solution, respectively. Au NPs with different modifications can interact with BSA and the hydrodynamic diameter or protein thickness increases determined by DLS and TEM [81]. Moreover, UV–vis–NR spectra can detect significant shifts in the peak position of the SPR of Au NPs before and after protein adsorption. The protein adsorption probably broadens the absorption spectra and decreases absorption intensity, which is a rapid and simple method to characterize the dynamic process of NP–protein interaction [83]. When human serum albumin (HSA) at a series concentration interacts with 2.95 nM citrate-coated Au NPs, the SPR peak intensity of Au NPs has a slight increase, along with a 2–4 nm red shift of the SPR peak, and a slightly broadened SPR band [84].

Based on the signals of left and right circularly polarized light, CD spectra are suitable to determine secondary structure of adsorbed proteins on Au NPs in aqueous solution [85]. When 100 *μ*g mL^−1^ BSA in phosphate-buffered saline (PBS) was mixed with Au NPs (at 0, 0.02, 0.04, 0.06, 0.08 and 0.10 *μ*g mL^−1^), in the formed BSA–Au NP conjugates, the percentage of *α*-helical structure significantly decreased. The structure of the *α*-helix has a characteristic CD signal in the far UV region. After conjugation, a significant decrease of ellipticity at the band around 208 nm and 220 nm was observed, which meant that the *α*-helical structure was destroyed and protein was unfolded after adsorption; when proteins are bound to Au NPs in biological fluids, the conformation changes with increasing protein concentration [86].

Furthermore, a quartz crystal microbalance (QCM) has been used to study the kinetics constants of adsorbed proteins quantitatively [87, 88]. Compared with SPR, QCM has a distinct advantage which can monitor multilayer corona, because the sensitivity of QCM was unchanged at the thickness of 400 nm, but the SPR peak distinctly broadened at 200 nm. When proteins are adsorbed on the surface of Au NPs, the surface plasmon waves will be changed as previously mentioned. For example, BSA, myoglobin (Mb), and cytochrome c (CytC)) can adsorb on mercaptoundecanoic acid (MUA)-capped Au NPs. QCM results showed that the adsorbed BSA and CytC are monolayer structure, however, Mb exists in the form of a bilayer. BSA has the highest affinity on Au NPs compared with Mb and CytC [87]. ITC is also a conventional analytical technique that can explore the thermodynamics parameters, such as binding stoichiometry, binding affinity, and binding enthalpy change. When BSA interacts with positively and negatively charged NPs, phosphonic acid-coated NPs have the highest affinity, supported by ITC study, while the amine-modified NPs (positively charged) adsorbed fewer proteins [89].

In addition, nuclear magnetic resonance (NMR) has been used to identify and quantify adsorbed proteins and characterize the structure of corona. When proteins adsorb on the surface of Au NPs, the SPR will be changed, leading to a red shift in the adsorption spectrum and changing the dielectric constant of the interface. The interaction changes the resonance angle of incident light and resonance wavelength, which is helpful for studying the dynamic processes of protein adsorption [90]. NMR has been used to study how different sizes of Au NPs (range from 10 to 30 nm) interact with human ubiquitin (hUbq). Each peak represents a NH group and the chemical shift is sensitive to the chemical environment. Based on the changes in the peaks of [15N-1H]-HSQC (heteronuclear singular quantum correlation) NMR spectra, the chemical shift was thus used to show the adsorbed protein structure on Au NPs after they were mixed with hUbq in a buffer [91].

Conventional analytical techniques like chromatography, capillary electrophoresis (CE) [92], one-dimensional electrophoresis (1D-E) [93], two-dimensional electrophoresis (2D-E) [94], and sodium dodecyl sulfate-polyacrylamide gel electrophoresis (SDS-PAGE) are useful for the isolation and identification of proteins in the corona composition. Polyacrylamide gel has a mesh structure with molecular sieve effect, and NP-bound proteins can be separated by SDS-PAGE. The protein mobility highly depends on its relative molecular weight, and is less correlated to the charge and molecular shape. García *et al* prepared Au NPs (Au14) that were stabilized by citrate, glycoconjugates of N-acetylglucosamine (GlcNAc), or PEG, and Au NPs that were coated with CTAB, disaccharide lactose, or PEG. They used SDS-PAGE to determine the amounts of bound proteins when Au NPs were present in physiological media. The conclusion was that charged NPs (capped with citrate or CTAB) would adsorb more proteins than those with neutral surfaces (stabilized with PEG or glycans) due to electrostatic interaction [95]. In addition, other analytical methods like mass spectrometry (MS) have been used to identify the adsorbed protein profile [96].

**Figure 3.**
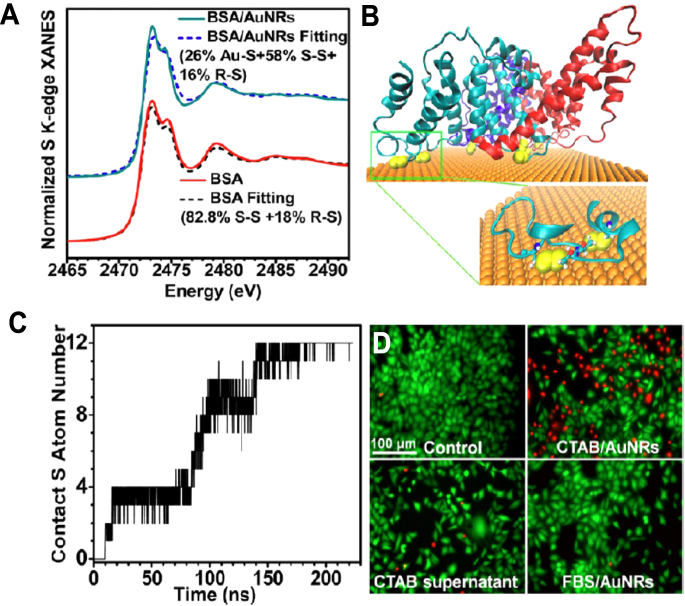
The binding structures of BSA protein to the surface of Au NRs and the influence on cytotoxicity. (a) The adsorption of BSA on Au NRs by Au-S bond, based on XANES. (b) Disulfides of BSA (yellow) binding to the Au (111) surface of AuNRs. BSA is rendered as a cartoon representation with the three domains colored cyan, red, and blue. Inset: zoomed, two disulfide moieties colored yellow on the surface of Au from the green segment. (c) Number of sulfur atoms in contact of an individual BSA on the gold surface accompanying with time based on molecular dynamics simulation. (d) Cytotoxicity of CATB/Au NRs or serum protein adsorbed CATB/Au NRs, determined by LIVE-DEAD assay. Reprinted with permission from [65]. Copyright 2013 American Chemical Society.

To investigate the bound interface structure of proteins, we used synchrotron radiation x-ray near edge absorption spectroscopy (XANES) to study how BSA is adsorbed on the Au NRs and measure the number of Au–S bonds after the protein are bound to NPs. The protein absorption on the surface was contributed by at least 12 Au–S bonds to be a stable corona (figure 3) [65]. Combining with sulfur-XANES, computer-assisted molecular dynamic simulation could help explore the binding sites and interfaces on the proteins. These methods will improve our understanding about what happens at NP–protein interfaces, which can provide a basic explanation about corona-mediated effects [97]. Novel methods or integrated methodology are urgently required to reveal the interfacial structure of corona and Au NPs. Promising techniques should not only realize high-throughput screening (HTS) and identification of proteins in various biological fluids, but also precisely predict potential biological effects quickly, with real time and high resolution.

## 3. Interaction of Au NPs with cells

Biological effects of nanomaterials are commonly modulated by several factors: the first is the physical and chemical properties of nanomaterials, particularly the surface properties. The second is the formation of the solid–liquid interface, referring to the interaction between nanomaterials and biological fluids. The third is the interplay between the solid–liquid interface and biological substrate. The interaction between Au NPs and cells is an important link between molecules and individual levels.

The cell membrane is an important barrier that is in charge of transporting and exchanging intracellular and extracellular substances. Physicochemical properties of Au NPs, including shape, size, aspect ratio, surface modification, and charges are closely related to their biological effects [98]. Au NPs in biological fluids could form NP–protein complexes that can be recognized by cell membrane receptors and then uptaken by cells. Au NPs can also be wrapped by the retracted cell membrane, and thus be directly transported into cells, which can directly affect the cellular responses [99]. Au NPs can be internalized by cells in different ways: receptor-mediated endocytosis and phagocytosis pathways (figure 2(b)). The processes in both pathways include the formation of Au NP–protein complexes, recognition by cell membrane receptors, engulfment into a vesicle by the cells, being transported or penetration into cells, the activation of signal pathways, sequential trafficking inside cells, and storage or elimination of Au NPs by cells [42]. For example, 2 and 6 nm Au NPs are distributed in the nucleus and the cytoplasm, while 15 nm Au NPs only exist in the cytoplasm [100]. In another work, a series of Au NPs have sizes ranging from 2.4 nm to 89 nm and have been modified with poly(ethylene glycol)-functionalized dithiolane ligands terminating in either carboxyl or methoxy groups and covalently conjugated to cell penetrating peptides. The results showed that smallest 2.4 nm Au NPs can enter into the nucleus, while Au NPs with medium sizes (5.5 and 8.2 nm) are distributed in the cytoplasm. In contrast, the 16 nm or larger AuNPs have a low uptake by cells and were located at the cellular periphery [99].

### 3.1. Factors to influence cellular effects of Au NPs

Cellular effects of Au NPs are the synergetic interactions of various factors, including dose, exposure time, the properties of NPs such as surface chemistry and net charge, size, modification, shape, or even protein corona. We will discuss how these factors affect cytotoxicity in detail below.

#### 3.1.1. Dose- and time-dependent effects

Dose and time are two basic factors involved in NP-induced cytoxicity. The reason is that a higher dose may cause more cellular uptake of Au NPs and induce longer effects on the cells. Time effect can be well understood; the persistent performance of some toxic NPs influences the physiological functions. For example, citrate-coated 13 nm Au NPs can cause dose- and time-dependent effects on human dermal fibroblasts (CF-31), including cell proliferation rate, disruption in the microfilament structures, and induced apoptosis. When CF-31 cells were treated with 13 nm Au NPs for 2 d at gradients of Au NP concentrations with 20, 40, 60, 80, 100, 120, and 140 *μ*g mL^−1^, the doubling time for cells lengthened from 36 h to 37.5 h, 41 h, 43 h, 45 h, and 47 h. For 4 d treatment with Au NPs, the doubling time for cells changed from 36 h to 41 h, 44 h, 49 h, 56 h, and 61 h. With respect to 6 d treatment with Au NPs, the doubling time for cells varied from 36 h to 44 h, 47 h, 58 h, 66 h, and 76 h. These results indicated that citrate-coated Au NPs can inhibit the cell growth rate associated with incubation time and concentration of NPs. Furthermore, both longer exposure time and higher dose of Au NPs induced greater apoptosis ratio. For 3 d exposure, the apoptotic ratios for cells were 0.3% for control, 23% at 95 *μ*g mL^−1^, 26% at 142 *μ*g mL^−1^, and 43% at 190 *μ*g mL^−1^, while for 6 d exposure, the apoptosis percentages were 60%, 80%, and 96%, respectively. The Au NPs also lead to microfilament disruption with changed cell aspect ratio, dependent on Au NP dosage [101]. It was also observed that citrate-coated Au NPs can inhibit the proliferation rates of human adipose-derived stromal cells in a dose-associated mode. Au NPs at 190 *μ*g mL^−1^ reduced cellular growth rate greater than those at 95 and 142 *μ*g mL^−1^ [102]. With respect to CTAB-capped Au NRs, they induced dose- and time-dependent effects on cytotoxicity. Au NRs can cause time- and dose-related cytotoxicity to cancer cell lines such breast cancer cell (MCF-7) and human lung adenocarcinoma cell (A549). The higher concentrations of Au NRs or the longer exposure time, the greater toxicity to cells were observed. The reason was that treatment at higher concentration or longer exposure can increase the cellular uptake of cytotoxic NPs and more accumulation of NPs, which caused stronger toxicity [31, 79].

#### 3.1.2. Size- and shape-dependent effects

Cytotoxicity of Au NPs is partly associated with the size and shape of NPs. Citrate-capped Au NPs of various sizes (5 and 15 nm) caused different toxicity to mouse fibroblasts cells during 72 h exposure. For example, the 5 nm Au NPs induced greater cytotoxicity than 15 nm ones. At 50 *μ*M, 5 nm Au NPs can induce greater toxicity than 15 nm Au NPs [103]. The size dependence in cytotoxicity of Au NPs was also observed for other cell lines including HeLa cells and SK-Mel-28 melanoma cells (SK-Mel-28), L929 mouse fibroblasts (L929), and mouse monocytes/macrophage cells (J774A1) [48, 104]. Moreover, the shape of Au NPs also influences cellular uptake and cytotoxicity. We have shown that the uptake and cytotoxicity of CTAB-coated Au NRs highly depended on the aspect ratio. The shorter Au NRs are easier to internalize than longer ones and also have a higher cytotoxicity [79]. The possible reason was that spherical or shorter NPs can be engulfed and internalized by mammalian cells more effectively than longer NRs [105]. Another work showed that CTAB-coated Au spheres (with a 43 ± 4 nm diameter) can cause greater toxicity than Au NRs (38 ± 7) × (17 ± 3) nm because CTAB-coated spheres may have a higher release of toxic CTAB upon intracellular accumulation that contributed to the higher toxicity [106]. In addition, the size of Au NPs influences cell proliferation, cellular uptake, cytoskeleton, cell shape, and apoptosis. Compared to 13 nm Au NPs, 45 nm citrate-coated Au NPs caused more apoptosis ratio that changed the cell shape or cell aspect ratio more dramatically. When Au NPs were internalized by cells, the number of vacuoles, rather than the absolute concentration of particles within the cells, plays the crucial role in disrupting normal cellular function and the induced cytotoxicity. During the uptake of 45 nm Au NPs, cells could generate more vacuoles, and then collapsed and released in the cytoplasm to damage cells [101].

#### 3.1.3. Surface chemistry

Surface chemistry is another factor to mediate the cellular effects of Au NPs. Generally, the surface modification is to increase their dispersion, stability, targeting. It is worth mentioning that the modification will change the net charges and surface properties of Au NPs that may influence the cellular effects.

Qiu *et al* studied the cytotoxicity of Au NPs modified with CTAB, PSS, or PDDAC. They found that CTAB-modified Au NPs are more toxic than PSS and PDDAC-modified ones to human breast cancer (MCF-7) cells. The possible reason was the intracellular CTAB-capped Au NPs may release CTAB molecules that will destroy the membrane of organelles such as lysosomes and mitochondria to induce cell apoptosis. Once further modified with PSS and PDDAC, the cytotoxity of Au NPs was significantly reduced [79]. Another study also showed that citrate-coated Au NPs induce apoptosis in human lung cancer A549 cells, but Au NPs coated with polyethylenimine (PEI) showed negligible toxicity to both A549 cells and MCF-7 cells [107]. Another study showed that the cytotoxicity of triphenylphosphine monosulfonate-modified Au NPs of 1.4 nm (Au1.4MS) and glutathione-modified NPs of 1.1 nm (Au1.1GSH) was different. They found that GSH modification significantly decreased cytotoxicity, in which the IC50 of Au1.1GSH (3130 *μ*M) is 65-fold higher than Au1.4MS (48 *μ*M) [103]. Furthermore, the polymer coatings on the Au NPs influence the endothelial cell uptake of NPs. The Au NPs with a mean size of 35 nm can be modified with positively-charged ethanediamine, neutrally-charged hydroxypropylamine, glucosamine, and poly (ethylene glycol). None of these Au NPs exhibited cytotoxicity even at a dose of 250 *μ*g mL^−1^. It was interesting that the positively-charged Au NPs had a greater cellular uptake than other Au NPs, while more Au NPs coated with hydroxypropylamine were internalized than other neutrally-charged NPs. The possible reason was that different coatings result in a different uptake route for Au NPs and the hydroxypropylamine-coated NPs may adsorb more proteins on the cell membrane to promote cell uptake. Compared with other NPs, the positively-charged NPs may prefer to interact with the extracellular matrix with the opposite charge, which promoted cell uptake [108]. Thus, surface coatings play crucial roles in inducing the cytotoxicity of NPs and cellular uptake.

Surface charge is another important factor to mediate cytotoxicity of Au NPs. The membrane is a lipid bilayer structure that contains a large amount of phosphate to make the cell membrane negatively charged. Positively charged Au NPs can be easily attracted to the cell membrane electrostatically and cause damage to the cell membrane. The positively charged NPs or cationic polymer could interact with the cell membrane to form NP-micelles, resulting in nanosized holes [109]. Au NPs with cationic side chains are easy to adsorb on the surface of cell membrane, which increases the cell membrane permeability and significantly reduces cell viability [110]. Moreover, the ligand coatings with trimethylammoniumethanethiol (TMAT), mercaptoethanesulfonate (MES), or mercaptoethoxyethoxyethanol (MEEE) on Au NPs provide positive, negative, and neutral charges, respectively. These Au NPs showed distinct toxicities to human keratinocytes (HaCaT): the positively and negatively charged Au NPs exhibit stronger toxicity at 10 *μ*g mL^−1^ than neutral ones [43]. In addition, amphiphilic polymer-grafted Au NPs have different surface charges. The positively charged Au NPs were more cytotoxic than negatively charged ones due to the electrostatic adsorption of Au NPs on the cell membrane, which may increase penetration into the cell membrane [111].

Furthermore, our work revealed that the assembly of ligands on the Au NP surface plays crucial roles in inducing cytotoxicity of Au NPs rather than surface charges. TEM, environmental scanning electron microscope (ESEM), lactic dehydrogenase (LDH) assay, and LIVE-DEAD assay were used to compare the cytotoxicity of Au NRs with different coatings such CTAB and PDDAC. PDDAC-coated Au NRs caused negligible toxicity to cells in serum-free media, while CTAB-coated Au NRs induced acute toxicity to cells by penetrating into the cell membrane and increasing cell membrane permeation to lead to necrosis. The reason is that CTAB molecules form bilayer structures on the Au NR surface and the CTAB bilayer is prone to be dissolved in lipid bilayers after Au NRs attach to the cell membrane [65, 112]. CTAB-coating may disrupt the membrane structures of the endosome/lysosomes and probably have a mitochondrial target to selectively inhibit the growth and migration of cancer cells [31, 113, 114].

### 3.2. Cellular responses

Generally speaking, the cellular responses to Au NPs include changed physiological functions, cell morphology, cell cycle and proliferation, differentiation and so on (table 1). An understanding of these adverse effects caused by Au NPs will help us design materials more rationally.

**Table 1.** Cellular responses induced by different types of Au NP.

Shape/size	Modification	Cell line	Uptake/location	Behavior/differentiation	Damage/toxicity	Molecules involved	Signal pathway	References
Rod 55 nm	Serum protein	A549, 16HBE MSC cells	Clathrin-mediated, from lysosome to mitochondria	Morphological change	Selective lysosomal membranes and actin damage	ROS	Mitochondrion-related pathway	[31]
Sphere 4 nm	PMA layer	HUVECs, C17.2 cells, PC12 cells	Active endocytosis	Cell cycle arrest	Cytoskeleton damage	Focal adhesion kinase, ROS	Actin-mediated pathway	[133]
Sphere, rod, urchin, 10–80 nm	PEG, CTAB	Microglia neural cells, transgenic mouse	Shape-dependent	N	Autophagy phagocytosis	IL-1 *α*, TLR-2 TNF-*α*, GM-CSF	Pro-inflammatory signals	[134]
Sphere 5, 10, 20 nm	PAA, PDHA	HL-60, HEK293 THP-1	N	N	Inflammation	Mac-1, IL-8, TNF-*α*	Mac-1 receptor pathway	[28]
Sphere 20 nm	Negative charged	MSCs osteoblast cells	Receptor-mediated endocytosis	MSCs toward osteoblast cells	Mechanical stress	p38	p38 MAPK pathway	[121]
NPs 20 nm	FBS	MRC-5 human lung fibroblasts	Endosomes, lysosomes	Autophagosome	Oxidative damage	ROS MAP-LC3	LKB1-AMPK signal pathway	[135]
Sphere 10, 25, 50 nm	Negatively charged	Normal rat kidney cells	Size-dependent endocytosis	N	Lysosome impairment	LC3, p62	Autophagic pathway	[35]
NPs 5, 20, 50, or 100 nm	Unmodified	EOC, A2780, OVCAR5, SKOV3-ip, OSE	Size-dependent endocytosis	Inhibition of tumor growth and metastasis	N	TSG-14, MMP 8, bFGF, TGF-*β*, E-cadherin, HB -GFs	p38-MAPK pathways, EMT	[136]
Au NRs	PSS, SiO_2_	NG108-15 neuronal cells	N	Differentiation	N	Ca^2+^	Ca^2+^ signal	[32]
Au NPs 2.7 nm	Tiopronin	MCF-7,HeLa L929, H520	Endosomes, lysosomes, perinuclear areas	N	Cytotoxicity depends on dose	ROS	N	[126]
Spherical Au NPs 21 nm	N	Male C57BL/6 mice,	Abdominal fat tissue, liver	N	No cytotoxicity	TNF*α*, IL-6	Inflammation related	[137]
A NR	Cetyltrimethylammonim bromide	A549, 16HBE	Mitochondria, lysosome	Metabolic change	Oxidative stress, mitochondria damage	Lactate GSH GSSG	Metabolic pathway	[113]
Au NRs 62.3 nm	PDDAC, PSS, PEG	MEF-1, MRC-5	Lysosome	N	Depend on dose and cell types	Bach-1, HO-1, ROS,	HO-1 pathways	[128]
Au NPs	PEG-silane layer, cRGD, PEG-diacrylate (PEG-DA) hydrogels	Hematopoietic KG-1a, REF52	N	Cell adhesion	Affect cell behavior	N	N	[130]
Au NPs	Single citrate capped	A549	Lysosome	Cycle arrest at the G0/G1 phase	Aggregation of the MTs, apoptosis	Bax, p53, Bcl-2, PARP	Apoptosis-related pathway	[118]

**N**: not mentioned. **EOC**: epithelial ovarian cancer. **OSE**: ovarian surface epithelial. **A549**: human alveolar adenocarcinoma epithelial cells. **16HBE**: normal bronchial epithelial cells. **MEF-1**: mouse embryo fibroblast cell line. **MRC-5**: human embryonal lung fibroblast cell line. **REF52**: rat embryonic fibroblast. **PDDAC**: poly (diallyldimethyl ammonium chloride). **PSS**: polyethylene glycol and polystyrene sulfonate. **HO-1**: heme oxygenase-1. **MTs**: microtubules. **PARP**: poly (ADP-ribose) polymer. **MSCs**: mesenchymal stem cells. **PMA**: a phorbol 12-myristate 13-acetate.

#### 3.2.1. Cell cycle arrest and DNA damage

The exposure to Au NPs may change the cell cycle and result in DNA damage. Jeyaraj *et al* found that 15 nm Au NPs can trigger human cervical carcinoma cell (HeLa) cycle arrest and DNA damage. These NPs were able to induce apoptosis by activating caspase pathways and inducing mitochondrial dysfunction [115]. Citrate-coated Au NPs with sizes of 5 nm and 15 nm inhibit the proliferation of human primary lymphocytes and murine macrophages (Raw264.7). The reason was that Au NPs are able to trigger damage in chromosomes and apoptosis [116]. Moreover, 30 nm PEG-capped Au NPs can be further modified by arginine–glycine–aspartic acid peptide (RGD) and nuclear localization signal (NLS) peptide (RGD/NLS–Au NPs). These Au NPs will cause DNA damage to cancer cells and result in cell cycle arrest and apoptosis, but have less negative impact on normal cells [117]. Au NPs can also cause the aggregation of microtubules (MTs) of A549 cells that will lead to cell cycle arrest at the G0/G1 phase and concomitant apoptosis, which depends on the size, concentration, and incubation time [118].

#### 3.2.2. Gene and protein expression

Exposure to Au NPs may modulate the gene or protein expression of the cells. In a recent study, macrophages have been treated with Au NPs of various sizes (3, 6, and 40 nm) for 24 h at concentrations between 1 and 10 *μ*g mL^−1^. After treatment, the cell morphology showed a spread shape, while untreated cells remained round. The exposure to Au NPs could also result in a significant up-regulation of the pro-inflammatory genes IL-1, IL-6 and TNF-*α* [26]. With respect to immune cells, Au NPs will down-regulate paired box 5 (pax5) and up-regulate the expression of B-lymphocyte-induced maturation protein 1 (blimp1) to promote the secretion of IgG to strengthen humoral immunity. As a result, the secretion of IgG highly depended on the size of Au NPs with a maximum efficiency at 10 nm [119]. Furthermore, the proteomic method has been applied to study the expression of protein levels after exposure to Au NPs. When mouse Balb/3T3 fibroblast cells were treated with 5 or 15 nm Au NPs, 88 and 83 proteins were modulated, respectively. Proteomic results revealed that both AuNPs trigger several pathways and associated proteins related to cell morphology, cellular function and maintenance, cellular growth and proliferation, cell cycle, oxidative stress, and inflammatory responses [120].

#### 3.2.3. Cell differentiation and signal pathways

Au NPs may induce cell differentiation. Studies have shown that Au NPs can interact with the receptor proteins in the cell membrane, activating the p-38 mitogen activated protein kinase pathway (MAPK) signaling pathway. As a result, Au NPs modulate the expression of differentiation-relevant genes that promote osteoblast differentiation from mesenchymal stem cells (MSCs) and inhibit their differentiation into adipocytes. The possible mechanism was that Au NPs may interact with the cell membrane and cytoplasmic proteins during cellular uptake. As a result, the uptake process might trigger mechanical simulation to cells and activate the p-38-MAPK signal pathway, leading to differentiation into osteoblasts [121]. Furthermore, the Au NPs internalized by pre-osteoblasts can markedly promote them to differentiate into osteoblasts. The reason was that the expression of BMP-2, Runx-2, OCN, and Col-1 were significantly up-regulated and the ERK/MAPK pathway was activated due to the exposure to Au NPs [122]. When NG108-15 neuronal cells were treated with PSS-or SiO_2_-coated Au NRs, the cells differentiated into neuron-like cells under NIR laser irradiation. A possible mechanism was that photons can change transients and the intracellular Ca^2+^ signaling that is highly involved in cell differentiation [32].

**Figure 4.**
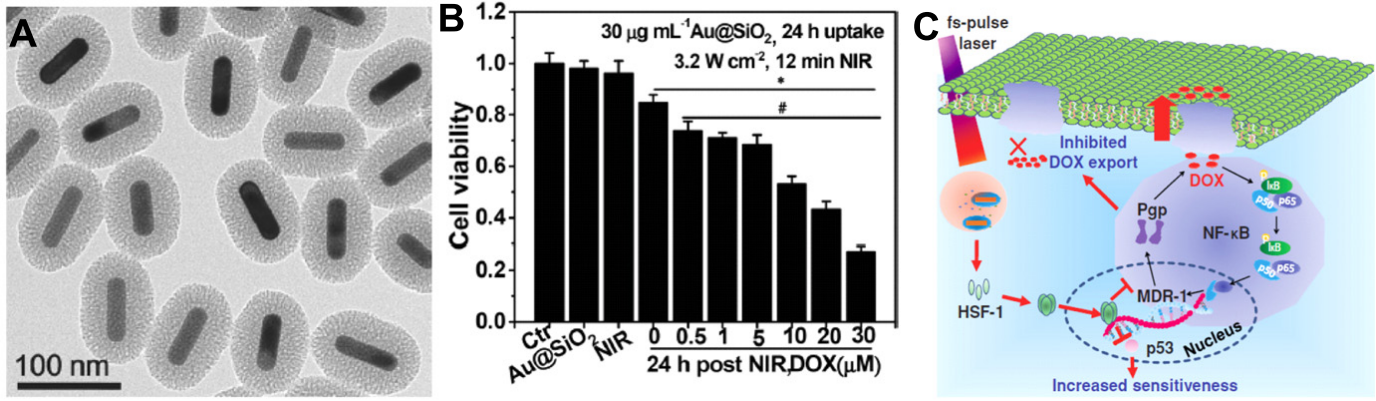
(a) TEM image of Au@SiO_2_ nanocarrier. (b) Influence of photothermal approach on cell sensitivity to DOX by a 780 nm fs-pulse laser irradiation at 3.2 W cm^−2^ for 12 min. Before NIR irradiation, MCF-7/ADR cells were exposed to 30 *μ*g mL^−1^ Au@SiO_2_ in cell culture medium for 24 h. (c) Mechanisms of the reversal of drug resistance of cancer cells under fs-pulse laser irradiation. Photothermal stimulus triggers the activation of heat shock factor (HSF-1) to depress NF-*κ*B pathway that dominates in the regulation in the characteristics of drug resistance. As a result, NIR irradiation modulates cell-signaling pathways to increase the sensitivity of MCF-7/ADR to doxorubicin (DOX) as well as to enhance the DOX accumulation. Reprinted with permission from [114]. Copyright 2014 WILEY-VCH Verlag GmbH & Co. KGaA, Weinheim.

Au NPs can also activate signal pathways for important cellular events. A previous study demonstrated that citrate-stabilized 10 nm Au NPs can induce the activation of the NF-*κ*B signaling pathway in the murine B-lymphocyte cell line (CH12.LX). This pathway is a way for Au NPs to mediate the inflammatory responses of lymphocytes [81]. We used mesoporous silica and Au NRs to construct a core–shell structure as a promising and multiple theranostic nanocarrier [10, 114]. Under NIR laser irradiation, the intracellular Au NRs can induce a medium elevation of local temperature that efficiently circumvented the drug resistance characteristics of cancer cells. We found that the local mild thermal stimulus and the triggered generation of free radicals by laser treatment enhanced the expression of heat shock factor-1 (HSF-1), which depressed the activation of NF-*κ*B pathways (figure 4) [114]. Importantly, the NF-*κ*B pathway is well known to regulate drug resistance using high expression of pump proteins in the cell membrane and low sensitivity to toxic chemotherapeutic drugs [114, 123]. As a result, local NIR irradiation can successfully depress resistant pathways to overcome drug resistance. Our recent work revealed that CTAB-capped Au NRs can regulate the expression of mitochondrial proteins and repress the pathways of glucolysis and the generation of energy based on proteomic techniques. As a result, these Au NRs are capable of suppressing the migration of cancer cells *in vitro* and metastasis *in vivo* [124].

#### 3.2.4. Reactive oxygen species (ROS) generation

Intracellular Au NPs may induce ROS generation. A study showed that surface modification of Au NRs may generate different ROS levels. PEG-capped Au NPs were used to incubate with three different types of cell lines: rat PC12 pheochromocytoma cells, murine C17.2 neural progenitor cells, and human umbilical vein endothelial cells (HUVECs). In a concentration dependent mode, Au NPs can decrease the mitochondrial membrane potentials, induce the generation of ROS, elevate intracellular Ca^2+^ levels, and cause DNA damage [125]. Another work showed that tiopronin-coated Au NPs (Au NPs-TP) could be internalized by cancer cells and mainly located in the endosomes and the lysosomes with a capability of inducing ROS generation, which was largely dependent on the exposure time, concentration of Au NPs, and cell types [126]. In addition, Au NPs can induce oxidative stress and endoplasmic reticulum stress, resulting in autophagy [127].

**Figure 5.**
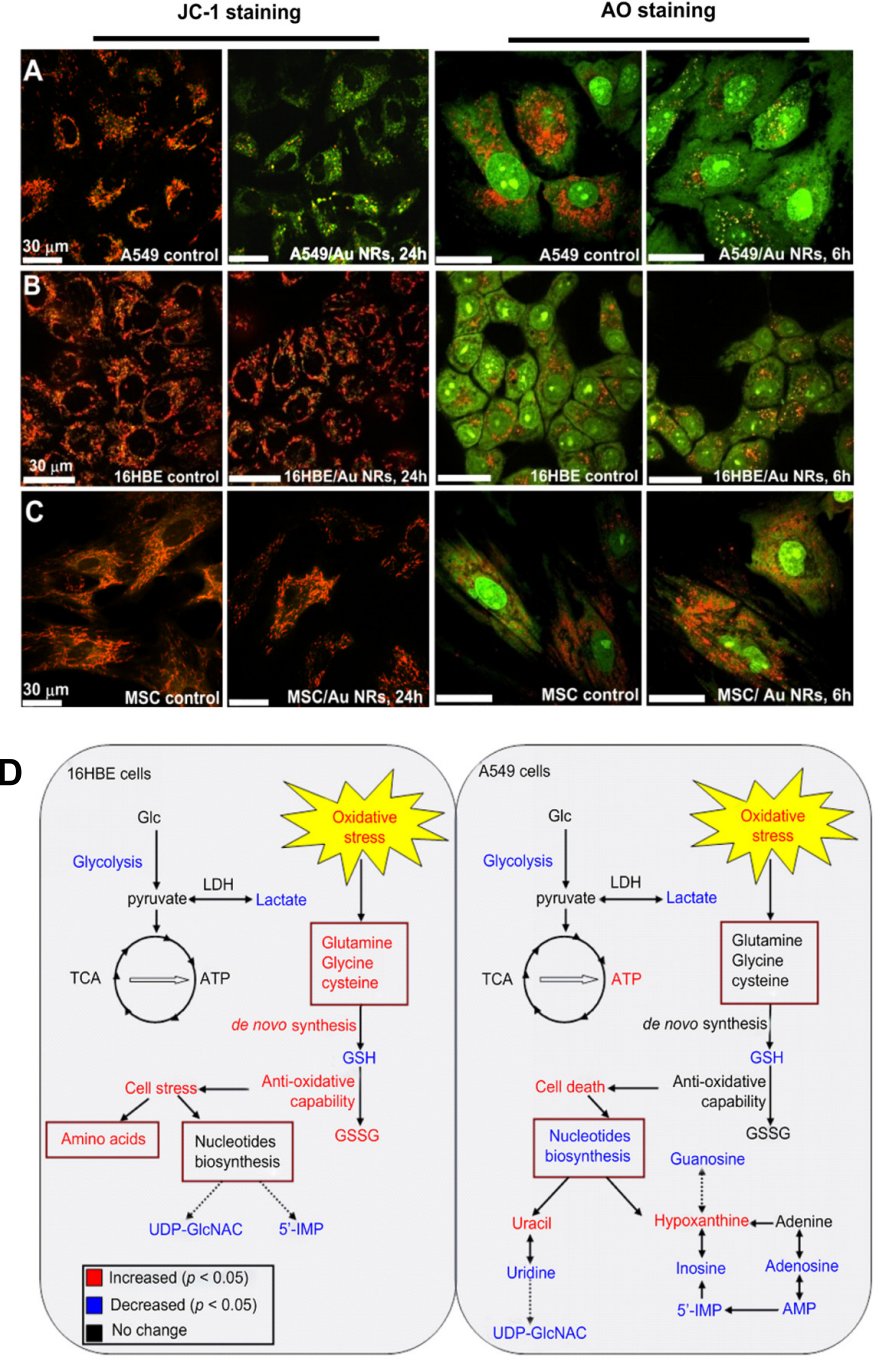
(a)–(c) Influence of serum protein-coated CTAB/Au NRs on the lysosomal membrane permeation by AO assay and the mitochondrial membrane potentials by JC-1 assay after they are exposed to 16HBE normal cells and A549 cancer cells. Reprinted with permission from [31]. Copyright 2011 American Chemical Society. (d) Schematic of metabolic responses of A549 and 16HBE cells when they are exposed to serum protein-coated CTAB/Au NRs, metabolites in red or blue indicate remarkable change in its level. Reprinted with permission from [113]. Copyright 2013 Elsevier Ltd. All rights reserved.

We found that CTAB-capped Au NRs can be engulfed by human normal bronchiolar epithelium cells (16HBE) and human lung cancer cells (A549). However, these Au NPs have a distinct destination in two kinds of cells. Au NRs can target mitochondria of A549 cells and largely decrease the mitochondrial membrane potentials, resulting in a high ROS level to induce apoptosis. But the localization of Au NRs in 16HBE was the lysosomes, in which Au NRs showed a smaller effect on free radical generation (figures 5(a)–(c)) [31]. Based on the metabonomic technique, we found that CTAB-coated Au NRs induced distinct effects on the cell viabilities of normal cells and cancer cells. Au NRs induced oxidative stress in both cells lines, but the normal cells are more able to offset the oxidative stress than the cancer cells with evidence of more conversion of GSH to GSSG in normal cells compared to cancer cells [113] (figure 5(d)). These Au NRs were able to induce oxidative stress and up-regulate associated proteins such as heme oxygenase-1 (HO-1) in MEF-1 but not MRC-5 cells [128].

#### 3.2.5. Cell morphology and adhesion

The exposure of Au NPs can also change the cell morphology. Incubating fibroblasts with citrate-coated Au NPs could induce the disappearance of stress fibers. These Au NPs could affect cell spreading and adhesion onto a culture substrate. They also inhibited the synthesis of extracellular matrix proteins. As a result, Au NPs repressed cell proliferation and significantly reduced cell viability [129]. Another work showed that Au NPs could influence the adhesive behavior of hematopoietic KG-1a and rat embryonic fibroblast (REF52) cells [130]. We also found that CTAB-coated Au NRs change the assembly of the actin cytoskeleton and inhibit the migration of cancer cells [31, 124].

## 4. Perspectives

Both the safety assessment and therapeutic efficiency of Au NPs are crucially important for their applications in biomedicine. For both aspects, it is necessary to address some basic questions about how the biological molecules or cells interact with Au NPs. One question is to know the factors influencing the cellular effects of Au NPs. Current studies have largely expanded our understanding about the biological effects of Au NPs, but detailed studies about the mechanisms are required. The complexity in the properties of NPs, the types of cells and microenvironments, and even physical factors influence the cell–NP interactions. It will be crucial to reveal how these factors mediate cellular effects. This new knowledge will benefit the rational design of nanomaterials in the future. The second question is the methodological challenge in studying NP–protein and NP–cell interactions. Proper methods are crucial to reveal how Au NPs affect structures and physiological functions of proteins and cells, and their fates. It is necessary to employ the state-of-art HTS techniques to predict potential risks to cells. Some -omics techniques are also powerful to reveal molecular details derived from mRNA, proteins, metabolites at the whole cell level. Combined with -omics and bio-information, researchers will elucidate signal networks to understand the feedback from cell–NP interactions. In addition, to capture the dynamic interaction processes, real time, sensitive, and single molecule-based analytical techniques will largely benefit the current research. The third question is how to capture the interfacial interaction information among NPs, proteins, and membrane structures *in situ* or in a live cell. Related research will provide detailed evidence about the chemical mechanisms for cellular effects of Au NPs. By resolving these problems, we can obtain more detailed information about their interactions and this knowledge will be helpful for the rational design of functional and biocompatible nanocarriers in the future.
